# Combining joint models for biomedical event extraction

**DOI:** 10.1186/1471-2105-13-S11-S9

**Published:** 2012-06-26

**Authors:** David McClosky, Sebastian Riedel, Mihai Surdeanu, Andrew McCallum, Christopher D Manning

**Affiliations:** 1 Department of Computer Science, Stanford University, Stanford, CA, USA; 2 Department of Computer Science, University of Massachusetts at Amherst, Amherst, MA, USA

## Abstract

**Background:**

We explore techniques for performing model combination between the UMass and Stanford biomedical event extraction systems. Both sub-components address event extraction as a structured prediction problem, and use dual decomposition (UMass) and parsing algorithms (Stanford) to find the best scoring event structure. Our primary focus is on *stacking *where the predictions from the Stanford system are used as features in the UMass system. For comparison, we look at simpler model combination techniques such as *intersection *and *union *which require only the outputs from each system and combine them directly.

**Results:**

First, we find that stacking substantially improves performance while intersection and union provide no significant benefits. Second, we investigate the graph properties of event structures and their impact on the combination of our systems. Finally, we trace the origins of events proposed by the stacked model to determine the role each system plays in different components of the output. We learn that, while stacking can propose *novel event structures *not seen in either base model, these events have extremely low precision. Removing these novel events improves our already state-of-the-art F1 to 56.6% on the test set of Genia (Task 1). Overall, the combined system formed via stacking ("FAUST") performed well in the BioNLP 2011 shared task. The FAUST system obtained 1st place in three out of four tasks: 1st place in Genia Task 1 (56.0% F1) and Task 2 (53.9%), 2nd place in the Epigenetics and Post-translational Modifications track (35.0%), and 1st place in the Infectious Diseases track (55.6%).

**Conclusion:**

We present a state-of-the-art event extraction system that relies on the strengths of structured prediction and model combination through stacking. Akin to results on other tasks, stacking outperforms intersection and union and leads to very strong results. The utility of model combination hinges on complementary views of the data, and we show that our sub-systems capture different graph properties of event structures. Finally, by removing low precision novel events, we show that performance from stacking can be further improved.

## Background

To date, most approaches to the BioNLP event extraction task [1,2] use a single model to produce their output. However, model combination techniques such as voting, stacking, and reranking have been shown to consistently produce higher performing systems by taking advantage of multiple views of the same data [3-6]. System combination essentially allows systems to regularize each other, smoothing over the artifacts of each (c.f. [7,8]). To the best of our knowledge, the only previous example of model combination for the BioNLP shared task was performed by [1]. Using a weighted voting scheme to combine the outputs from the top six systems, the task organizers obtained a 4% absolute F1 improvement over the best system used in isolation.

In this paper, we explore several model combination strategies. We aim to uncover the answers to these related questions: Which strategies are effective and why? How do the outputted event structures change after performing model combination? Finally, are there systematic errors which can be corrected to improve performance further?

We show that using a straightforward model combination strategy on two competitive systems (*base models*) produces a new system with substantially higher accuracy. This is achieved with the framework of stacking: a *stacking *model uses the output of a *stacked *model as additional features. To put the results in perspective, we also experiment with two simpler model combination techniques where systems are run independently and their outputs are combined via *union *or *intersection*.

Our base models are the UMass [9] and Stanford [10] event extractors. We initially considered combining these models using voting and reranking strategies. However, it seemed that given the performance gap between the two models, the best option was to include the predictions from the Stanford system into the UMass system (e.g., as in [7]). This has the advantage that one model (UMass) determines how to integrate the outputs of the other model (Stanford) into its own structure. In the case of reranking or voting, the combined model is required to output a structure constructed from the structures produced by the input models. In other words, each portion of the resulting structure originates from at least one of the base models. With stacking, one model determines how to integrate the outputs of the other model and the resulting structure can contain novel constructions. However, as it turns out, these novel constructions have low precision in our case. This can be understood using the same intuition that underlies the voting or union strategies - if a structure has been produced by multiple independent models, it is more likely to be correct. Novel events resulting from stacking have essentially been produced by neither base model and thus tend to be inaccurate. We show that by removing these novel events from our output, our state-of-the-art results can be improved further.

### The BioNLP shared task

**Figure 1:**
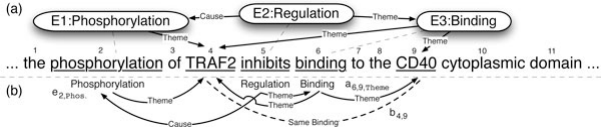
**Example of BioNLP events**. (a) The sentence, "... the phosphorylation of TRAF2 inhibits binding to the CD40 cytoplasmic domain ...," with target event structure to extract; (b) projection to a set of labeled graphs over tokens for the UMass model. The example includes three events, *Phosphorylation *(anchored by the text "phosphorylation"), *Regulation *(anchored by "inhibits"), and *Binding *(anchored by "binding"). Each event takes entities (proteins such as "TRAF2" or "CD40") or other events as arguments (THEME and CAUSE). In part (a), events are represented as rounded rectangles, relations as labeled arrows, and event anchors as dashed lines. In part (b), event types are written below their event anchors and relations are shown as labeled arrows or dashed lines (in the case of the "same binding" relationship).

The BioNLP shared task involves extracting a set of biomolecular events from natural language text in a given document (typically an abstract from a biomedical journal). By biomolecular events, we mean a change of state of one or more biomolecules. More concretely, let us consider part (a) of Figure 1. We see a snippet of text from a biomedical abstract and the three events that can be extracted from it. We will use these to characterize the types of events we ought to extract, as defined by the BioNLP 2009 and 2011 shared tasks. Note that for the shared task, entity mentions (e.g., proteins) are given by the task organizers and hence do not need to be extracted.

The event E1 in the figure refers to a *Phosphorylation *of the TRAF2 protein. It is an instance of a set of *simple events *that describe changes to a single gene or gene product. Other members of this set are: *Gene expression*, *Transcription*, *Localization*, and *Catabolism*. Each of these events has to have exactly one THEME, the protein whose state change is described. A labeled edge in Figure 1a shows that TRAF2 is the THEME of E1.

Event E3 is a *Binding *of TRAF2 and CD40. *Binding *events are special in that they may have more than one THEME, as there can be several biomolecules associated in a binding structure. This is in fact the case for E3.

In the top-center of Figure 1a we see the *Regulation *event E2. Such events describe regulatory or causal relations between events. Other instances of this type of events are: *Positive Regulation *and *Negative Regulation*. Regulations must have exactly one THEME; this THEME can be a protein or, as in our case, another event. *Regulation*s may also have zero or one CAUSE arguments that denote events or proteins which trigger the *Regulation*.

In the BioNLP shared task, we are also asked to find *anchor *(sometimes called *trigger *or *clue*) tokens for each event. These tokens ground the event in text and allow users to quickly validate extracted events. For example, the anchor for event E2 (a *Regulation *event) is "inhibit," as indicated by a dashed line.

**Figure 2:**
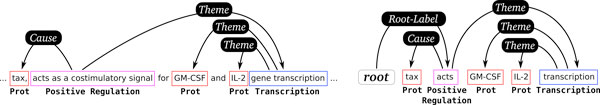
**Example of Stanford representation**. Event structures for the text fragment "... tax, acts as a costimulatory signal for GM-CSF and IL-2 gene transcription ..." (left) and the reduced form used internally in the Stanford model (right). In the reduced form, words that don't take part in any events (e.g., "for" and "and") are removed and multiword anchors are replaced with their syntactic heads (e.g., "acts as a costimulatory signal" becomes "acts").

Instead of directly working with the event representation in Figure 1a, both the UMass and Stanford systems extract labeled graphs in the form shown in Figure 1b. The vertices of this graph are the anchor and protein tokens. A labeled edge from an anchor *e *to a protein token *p *with role label *r *indicates that there is an event with anchor *e *for which the protein *p *plays the role *r*. An edge with role *r *from anchor *e *to anchor *e*' means that there is an event at *e*' that plays the role *r *for an event at *e*. This representation is used by the UMass system to define extraction as a compact optimization problem. A related representation is used by the Stanford system to tackle extraction as dependency parsing (see the Stacked Model section for details). If a graph can be drawn on a plane without crossing edges, we say that the graph is *projective *(sometimes referred to as a *planar graph*). Figure 2 shows examples of projective graphs while Figure 1 contains an example of a non-projective graph. We define the *non-projectivity *of a graph as the number of crossing edges in it. For more details about mapping back and forth between events and labeled graphs, we point the reader to [9,11,12].

The BioNLP 2009 shared task [1] consists of a single domain, Genia (GE) while the BioNLP 2011 shared task [2] expands the Genia domain and adds two additional domains, Epigenetics and Post-translational Modifications (EPI) and Infectious Diseases (ID) ([13-15], respectively). Our experiments in this paper are over the 2011 shared task corpora.

### Model combination approaches

Our primary approach consists of a *stacking model *that uses the predictions of a *stacked model *as features. In the following sections, we briefly present both the stacking and the stacked model and some possible ways of integrating the stacked information. We also describe two simpler model combination techniques (intersection and union) for comparison.

### Stacking model

As our stacking model, we employ the UMass extractor [16]. It is based on a discriminatively trained model that jointly predicts anchor labels, event arguments and protein pairs in bindings. We will briefly describe this model but first introduce three types of binary variables that will represent events in a given sentence. Variables *e_i,t _*are active if and only if the token at position *i *has the label *t*. Variables *a_i,j,r _*are active if and only if there is an event with anchor *i *that has an argument with role *r *grounded at token *j*.

In the case of an entity mention, this means that the mention's head is *j*. In the case of an event, *j *is the position of its anchor. Finally, variables *b_p,q _*indicate whether or not two entity mentions at *p *and *q *appear as arguments in the same *Binding *event.

Two parts form our model: a scoring function, and a set of constraints. The scoring function over the anchor variables **e**, argument variables **a **and *Binding *pair variables **b **is

$$s(e,a,b)\underline{\underline{\text{def}}}\sum_{e_{i},t=1}s_{\text{T}}(i,t)+\sum_{a_{i,j,r}=1}s_{\text{R}}(i,j,r)+\sum_{b_{p,q}=1}s_{\text{B}}(p,q)$$

with local scoring functions $s_{\text{T}}(i,t)\underline{\underline{\text{def}}}\langle w_{\text{T}},f_{\text{T}}(i,t)\rangle$, $s_{\text{R}}(i,j,r)\underline{\underline{\text{def}}}\langle w_{\text{R}},f_{\text{R}}(i,j,r)\rangle$ and $s_{\text{B}}(p,q)\underline{\underline{\text{def}}}\langle w_{\text{B}},f_{\text{B}}(p,q)\rangle$.

Our model scores all parts of the structure in isolation. It is a joint model due to the nature of the constraints we enforce: First, we require that each active event anchor must have at least one THEME argument; second, only *Regulation *events (or *Catalysis *events for the EPI track) are allowed to have CAUSE arguments; third, any anchor that is itself an argument of another event has to be labeled active, too; finally, if we decide that two entities *p *and *q *are part of the same *Binding *(as indicated by *b_p,q _*= 1), there needs to be a *Binding *event at some anchor *i *that has *p *and *q *as arguments. We will denote the set of structures (**e**, **a**, **b**) that satisfy these constraints as .

Stacking with this model is simple: we only need to augment the local feature functions **f**_T _(*i*, *t*), **f**_R _(*i*, *j*, *r*) and **f**_B _(*p*, *q*) to include predictions from the systems to be stacked. For example, for every system *S *to be stacked and every pair of event types (*t*', *t_S_*) we add the features

$$f_{S,t^{\prime },t_{S}}\left(i,t\right)=\left\{\begin{array}{cc} 1 & h_{S}\left(i\right)=t_{S}\wedge t^{\prime }=t\\ 0 & \text{otherwise} \end{array}\right)$$

to **f**_T _(*i*, *t*). Here *h_S _*(*i*) is the event label given to token *i *according to *S*. These features allow different weights to be given to each possible combination of type *t*' that we want to assign, and type *t_S _*that *S *predicts.

Inference in this model amounts to maximizing *s *(**e**, **a**, **b**) over . Our approach to solving this problem is dual decomposition [17,18]. This technique exploits the fact that while inference in the full problem may be intractable, it usually contains tractable subproblems for which efficient optimization algorithms exist. In dual decomposition, these algorithms are combined in a message passing scheme that often finds the global optimum of the full model. When a global optimum is found, dual decomposition also provides guarantees that prove the optimality of this solution.

For our event extraction model we divide the argmax problem into three subproblems: (1) finding the best anchor label and set of outgoing edges for each candidate anchor; (2) finding the best anchor label and set of incoming edges for each candidate anchor; and (3) finding the best pairs of entities to appear in the same *Binding*. For all of these problems, efficient algorithms can be derived [9].

For learning the parameters **w **of this model, we employ the online-learner MIRA [19]. MIRA iterates over the training data and compares the gold solution with the current best solution according to **w**. If both solutions disagree, **w **is adapted such that the gold solution would win with sufficient margin if the problem was to be solved again. We refer the reader to [16] for further details on both inference and learning.

### Stacked model

For the stacked model, we use a system based on an event parsing framework [10,20] referred to as the Stanford model in this paper. A high level description of the system relevant to the experiments in this paper follows. To train the Stanford model, first event structures are projected to dependency trees in a process similar to that in Figure 1b. These dependency trees are tree-rooted dependency graphs where nodes are event anchors or entities and the labeled, directed edges are relations, e.g., THEME and CAUSE. This projection eliminates some of the more complex aspects of event structures which cannot be captured easily in dependency trees, primarily events or entities with multiple parents. Words that do not take part in any events are removed in the dependency trees and multiword anchors of events are replaced with their syntactic heads. An example of this conversion can be seen in Figure 2.

After conversion, the dependency trees are parsed using an extension of MSTParser [21,22] which includes event parsing-specific features. To parse, MSTParser creates a complete graph with entities and event anchors as nodes. For each edge in the complete graph, MSTParser assigns a score using the features along that edge and the feature weights learned from training. At this point, the highest scoring parse (a subgraph of the complete graph which forms a tree) can be *decoded *using several possible algorithms. For example, the algorithm that gives MSTParser its name is the maximum-spanning tree algorithm which searches for a tree that spans all nodes in the graph and obtains the highest sum of edge scores. Once parsed, the resulting dependency tree is converted back to event structures. Training MSTParser involves learning feature weights which separate correct edges from incorrect edges during parsing.

Of particular interest to this paper are the four possible decoders in MSTParser since they result in four different models. These decoders come from combinations of feature order (first or second) and whether the resulting dependency graph is required to be projective. First-order features are features taken from a single edge (including the nodes at each end of the edge) while second-order features include features over two adjacent siblings along with their parent. Non-projective decoders would seem to be useful for this task. In Genia, 20.8% of the documents contain at least one non-projective arc (7.9% of the sentences and 2.9% of the overall dependencies [10]). This portion of the data can only be captured by non-projective decoders.

For brevity, the second-order non-projective decoder is abbreviated as '2N', first-order projective decoder as '1P,' etc. When referring to Stanford models, we always specify its decoder. Each decoder presents a slightly different view of the data and thus has different model combination properties. Projectivity constraints are not captured in the UMass model so these decoders incorporate novel information. Drawing on techniques from statistical constituency parsing [23,24], we employ a reranking framework to further improve performance and capture global features of event structures. The existing features are restricted to functions of a single edge in the first-order model and two adjacent siblings in the second-order model. However, some phenomena of event structures span larger structures (e.g., event anchors and all their immediate children or the number of THEME relations attached to a specific event). To switch to a reranking framework, we extend the decoders to *n*-best decoders which return the *n *highest scoring parses for each sentence (an *n*-best list) rather than just the single highest scoring parse. Note that our non-projective decoders have only approximate *n*-best decoders (exact inference for the 2N decoder is NP-complete [25]) resulting in suboptimal reranker models in some cases. The reranker rescores each parse in the *n*-best list and returns the highest scoring parse. These scores are based on features of the global event parsing structure as well as including metadata about the parse (e.g., the MSTParser's parsing score). The reranker can be also used for model combination when given the output from multiple *n*-best lists. In this case, unique parses are merged and the original number of decoders producing the parse and the scores from the decoders are added to the parse's metadata. While the primary focus of this paper is on using stacking for model combination, a small number of experiments study the performance of using the reranker for model combination.

#### Using the Stanford model as a stacked model

The projective Stanford models are helpful in a stacking framework since they capture projectivity which is not directly modeled in the UMass model. Of course, this is also a limitation since actual BioNLP event graphs are DAGs, but the Stanford models perform well considering these restrictions. Additionally, this constraint forces the Stanford model to provide different (and thus more useful for stacking) results.

To produce stacking output from the Stanford system, we need its predictions on the training, development and test sets. For predictions on the test and development sets, we used models learned from the complete training set. Predictions over training data were produced using cross-validation. Obtaining predictions in this way helps to avoid scenarios in which the stacking model learns to rely on high accuracy at training time that cannot be matched at test time.

We used 19 cross-validation training folds for GE, 12 for EPI, and 17 for ID. To produce predictions over the test data, we combined the training folds with 6 development folds for GE, 4 for EPI, and 1 for ID.

Note that, unlike Stanford's individual submission in the BioNLP 2011 shared task [26], the stacked models in this paper do not use the reranker. This is because it would have required making a separate reranker model for each cross-validation fold.

Training the stacking model took about two hours on a 16 core machine. The stacked model needed about three hours on a single core machine for each fold. Since the stacking model and each fold of the stacked model can be trained in parallel, the overall training time is about five hours if sufficient cores are available.

### Intersection and union

We investigate two baseline techniques for model combination: intersection and union. Both of these are similar to their standard set theory operations except that instead of using strict equality for events, we allow events to be equal if they match according to the BioNLP approximate recursive scoring metric.

## Results

Table 1 gives an overview of our results on the test sets on each of the four tasks we submitted to. Note that for the EPI and ID tasks we show the CORE metric next to the official FULL metric. The former is suitable for our purposes because it does not measure performance for negations, speculations and cellular locations--all of these we did not attempt to predict. Throughout this article, the notation *L *← *R *indicates that predictions from model *R *were used as stacking input to model *L*.

**Table 1:** Overall results

			UMass			Stanford			FAUST	
		Recall	Precision	F_1_	Recall	Precision	F_1_	Recall	Precision	F_1_
GE	Task 1	48.5	64.1	55.2	42.4	61.1	50.0	49.4	64.8	56.0
GE	Task 2	43.9	60.9	51.0	--	--	--	46.7	63.8	53.9

EPI	FULL	28.1	41.6	33.5	26.6	37.9	31.2	28.9	44.5	35.0
EPI	CORE	57.0	73.3	64.2	56.9	70.2	62.8	59.9	80.3	68.6

ID	FULL	46.9	62.0	53.4	46.3	55.9	50.6	48.0	66.0	55.6
ID	CORE	49.7	62.4	55.3	49.2	56.4	52.5	50.8	66.4	57.6
										
		FAUST (without novel)								
		Recall	Precision	F_1_						

GE	Task 1	47.6	69.7	56.6						

Results on test sets of all tasks we submitted to, for three models. We list recall, precision, and F_1 _using the standard BioNLP approximate recursive metric. For the GE and ID datasets, the Stanford model used all four decoders with the reranker. For EPI, the Stanford model used only the 1N decoder with the reranker. In all three domains, the stacked UMass←Stanford model (FAUST) used all four decoders from the Stanford model as inputs. The "FAUST (without novel)" is created by removing all events which don't occur in either the UMass or Stanford models (i.e., events which are novel to the stacked output).

We compare the UMass and Stanford standalone systems to the UMass←Stanford model (referred to as FAUST). This model uses the four Stanford predictions (1N, 1P, 2N and 2P) as stacking inputs to the UMass model. For all four tasks we observe substantial improvements due to stacking. The increase is particularly striking for the EPI track, where stacking improves F_1 _over the UMass model by more than 4.0 points on the CORE metric.

To analyze the impact of stacking further, Table 2 shows a breakdown of our results on the Genia development set. Presented are F1 scores for simple events, *Binding *events, *Regulation *events, and overall performance. We compare the standalone UMass system, various standalone Stanford models and stacked versions of these (UMass←*X*). The last line, "UMass←Stanford (all) without novel events," will be covered later in the discussion section.

**Table 2:** Stacking experiments on Genia

System	Simple	Binding	Regulation	Overall
UMass	74.7	47.7	42.8	54.8
Stanford (1N)	71.3	44.2	35.6	49.9
Stanford (1P)	70.7	42.6	34.6	49.1
Stanford (2N)	69.0	40.5	30.8	46.5
Stanford (2P)	72.0	40.3	35.4	49.5
Stanford (1N, reranked)	71.7	46.9	35.4	50.2
Stanford (1P, reranked)	70.6	44.5	35.5	49.4
Stanford (2N, reranked)	70.2	44.6	38.2	47.9
Stanford (2P, reranked)	71.1	47.8	35.9	50.5
Stanford (all, reranked)	71.6	**48.1**	35.9	50.7

UMass←Stanford (all) (= **FAUST**)	76.9	43.5	44.0	55.9
UMass←Stanford (1N)	76.4	45.1	43.8	55.6
UMass←Stanford (1P)	75.8	43.1	**44.6**	55.7
UMass←Stanford (2N)	74.9	42.8	43.8	54.9
UMass←Stanford (2P)	75.7	46.0	44.1	55.7

UMass←Stanford (all, anchors)	76.4	41.2	43.1	54.9
UMass←Stanford (all, arguments)	76.1	41.7	43.6	55.1

UMass←Stanford (all) without novel events	**77.3**	44.5	43.5	**56.2**

BioNLP F_1 _scores on the development section of the Genia track (task 1) for several event categories and overall F_1_. The best scores from each column are bolded.

Remarkably, while there is a 5 point gap between the best individual non-reranked Stanford system (1N) and the standalone UMass systems, integrating the Stanford 1N prediction still leads to an F_1 _improvement of 1 point. This can be seen when comparing the UMass, Stanford (1N decoder), and FAUST results. We also note that stacking the projective Stanford (1P) and Stanford (2P) systems helps almost as much as stacking all four Stanford systems combined. Notably, both Stanford (1P) and Stanford (2P) do not do as well in isolation when compared to the Stanford (1N) system. When stacked, however, they do slightly better. This suggests that projectivity is a missing aspect in the UMass system. This is explored in detail in the discussion section.

The "UMass←Stanford (all, anchors)" and "UMass←Stanford (all, arguments)" lines represent experiments to determine whether it is useful to incorporate only portions of the information from the Stanford system. UMass←Stanford (all, anchors) adds only the anchor predictions from the four Stanford models and does not significantly improve over the standalone UMass system. UMass←Stanford (all, arguments) includes only links between event anchors and their arguments and achieves a small improvement over the standalone UMass system but with scores significantly lower than when all information is stacked. Given the small gains for both of these over the original UMass system, it is clear that stacking information is most useful when attached to both anchors and arguments. Our theory is that most of our gains come from when the UMass and Stanford systems disagree on anchors and the Stanford system provides not only its anchors but also their attached arguments to the UMass system. Otherwise, the UMass system has little incentive to use the newly proposed anchors. This is supported by a pilot experiment where we trained the Stanford model to use the UMass anchors and saw no benefit from stacking (even when both anchors and arguments were used).

We provide a breakdown of our results on the Epigenetics track in Table 3, this time in terms of recall, precision, and F_1_. As before, there is a substantial boost in performance from stacking. The UMass model obtains an F_1 _of 63.9% on its own and jumps to 67.1% when given all four Stanford decoders as stacking input. For this dataset, the non-reranked Stanford decoders are not as far behind the UMass system, with the 1N decoder only 1% lower than the UMass system. When combined with a reranker, the Stanford model (1N decoder) even outperforms the UMass model. However, it is significantly more difficult to use the output of the reranked Stanford model as stacking input. Stacking would require nested cross-validation to produce outputs from the reranker over the training set. This is because training the reranker itself requires running cross-validation over the training set. Consequently, we were unable to perform experiments stacking the UMass model with the outputs from the reranked Stanford models. The "Stanford (1N+2P, reranked)" line shows an experiment where only the 1N and 2P decoders are used as input to the reranker. The hope was that the best single model (1N) would be improved by adding the most complementary decoder (2P) but the net result does not outperform the 1N model.

**Table 3:** Stacking experiments for the EPI track

System	Recall	Precision	F_1_
UMass	56.7	73.2	63.9
Stanford (1N)	52.2	79.0	62.9
Stanford (1P)	51.7	78.2	62.3
Stanford (2N)	48.1	**82.6**	60.8
Stanford (2P)	51.9	77.6	62.2
Stanford (1N, reranked)	57.7	73.2	64.6
Stanford (1P, reranked)	57.6	70.4	63.3
Stanford (2N, reranked)	55.3	75.4	63.8
Stanford (2P, reranked)	56.9	71.3	63.3
Stanford (1N+2P, reranked)	**57.9**	71.1	63.8
Stanford (all, reranked)	57.0	73.1	64.1
UMass←Stanford (all) (= **FAUST**)	**57.9**	79.7	**67.1**

BioNLP F_1 _scores on the development set of EPI using the CORE metric.

Table 4 shows our results on the development set of the ID task. Here the gap between Stanford-only results and the UMass results is much smaller and in some cases negligible. This seems to lead to more substantial improvements for stacking: UMass←Stanford (all) obtains an F_1 _2.2 points larger than the standalone UMass system. Also note that the projective systems do worse on their own, but are more useful when stacked. An unusual result is that for both EPI and ID (Tables 3 and 4) the Stanford (2N) decoder provides the highest precision but suffers in recall, resulting in a lower overall F_1_. This is because the 2N decoder ultimately ends up proposing fewer events (339 proposed events for EPI instead of ≈385 from the other three decoders).

**Table 4:** Stacking experiments for the ID track

System	Recall	Precision	F_1_
UMass	46.2	51.1	48.5
Stanford (1N)	46.9	50.2	48.5
Stanford (1P)	44.4	47.7	46.0
Stanford (2N)	45.0	**54.8**	49.4
Stanford (2P)	46.6	49.2	47.8
Stanford (1N, reranked)	47.5	51.4	49.4
Stanford (1P, reranked)	47.9	49.2	48.5
Stanford (2N, reranked)	45.7	52.3	48.8
Stanford (2P, reranked)	**49.6**	49.9	49.8
Stanford (all, reranked)	48.9	51.6	50.2

UMass←Stanford (1N)	45.8	51.6	48.5
UMass←Stanford (1P)	47.6	52.8	50.0
UMass←Stanford (2N)	45.4	52.4	48.6
UMass←Stanford (2P)	49.1	52.6	**50.7**
UMass←Stanford (all)	47.6	54.3	**50.7**
UMass←Stanford (2P, Conj)	48.0	53.2	50.4

Results on the development set for the ID track.

Another possible approach to stacking *conjoins *all the original features of the stacking model with the predicted features of the stacked model. The hope is that this allows the learner to give different weights to the stacked predictions in different contexts. However, incorporating Stanford predictions by conjoining them with all features of the standalone UMass system (UMass←Stanford (2P, Conj) in Table 4) does not help here. It appears that the increased data sparsity from the larger number of features ends up hurting performance.

In Table 5, we show a number of different model combination techniques for UMass and Stanford models. The top table summarizes relevant results on Genia from previous tables. The second table shows the results of using the baseline model combination techniques to combine Stanford results with the standalone UMass model. For each Stanford model, we show its independent performance as well as the effect of intersecting or unioning it with the output from the UMass model. None of these methods significantly improve performance above UMass's standalone performance on this dataset (54.7%). Unsurprisingly, the intersected results have significantly higher precision while the unioned results have significantly higher recall.

**Table 5:** Stacking and other model combination techniques

Model	F_1_		
		
UMass	54.7		
UMass←Stanford	55.8		
			
Model	Alone	Intersection with UMass	Union with UMass
Stanford (1N)	49.9	49.0	54.7
Stanford (1P)	49.0	48.3	54.6
Stanford (2N)	46.5	45.4	54.8
Stanford (2P)	49.5	49.1	54.4
Stanford (all)	--	42.4	53.0
Stanford (1N, reranked)	50.2	49.7	54.4
Stanford (1P, reranked)	49.4	50.2	53.2
Stanford (2N, reranked)	47.8	46.9	54.6
Stanford (2P, reranked)	50.4	50.0	54.4
Stanford (all, reranked)	50.7	50.0	54.7
			
Model		Intersection	Union

Stanford (all)		43.9	50.2

Stacking and reranking outperform the intersection and union model combination baselines. The first section of the table summarizes the results from the UMass and stacked models. The second section gives the performance of each Stanford model alone and when combined with the pure UMass model via the intersection and union methods. In the last section, we evaluate the intersection and union baselines using only the four Stanford models as inputs. The "Stanford (all)" line represents using all four individual decoders without model combination (hence the Alone column in the second table is left empty--it cannot be evaluated since it isn't a single set of outputs). In "Stanford (all, reranked)", the reranker was used to combine the four decoders into a single output before being intersected or unioned. All results are on the development set for the Genia track.

One additional form of model combination is shown in the "Stanford (all, reranked)" line in Table 5, bottom table. Recall that the reranker itself can be used to combine the outputs of multiple models. Allowing the reranker to choose the best events from the *n*-best lists from all four decoders yields an F_1 _of 50.7%. Combining the four decoders instead via unioning (bottom table) yields an F_1 _of 50.2% whereas combining them using the intersection baseline gives an F_1 _of 43.9%. Thus, reranking also improves over the union and intersection baselines. Creating a direct comparison of stacking versus reranking for performing model combination is left as future work.

We observed that the ID and GE corpora were similar in their annotations. This allowed us to apply techniques from domain adaptation. Our hypothesis was that it might be possible to augment the training data of the smaller ID corpus with the training and development data from the larger GE corpus. Merging both training sets is reasonable since there is a significant overlap between both in terms of events as well as lexical and syntactic patterns to express these. When building our training set we add each training document from GE once, and each ID training document multiple times--this lead to substantially better results than including ID data only once. For the UMass system, two copies of ID were used whereas the optimal performance for the Stanford system came from using three copies. Experiments on the Stanford parser with the 2N decoder can be seen in Table 6. While performance initially dips after adding GE data, once enough ID data has been added to adjust the distribution, the overall F_1 _improves. With three copies of GE, gains occur primarily in recall (38.0% to 45.0%) since the additional data helps to recognize additional patterns. Precision drops from 59.3% to 54.8% since the data from GE is not exactly from the same domain.

**Table 6:** Incorporating Genia data with training data for ID track

Model	Recall	Precision	F_1_
ID	38.0	**59.3**	46.3
ID (×1) + GE	40.2	52.0	45.3
ID (×2) + GE	41.7	52.4	46.4
ID (×3) + GE	**45.0**	54.8	**49.4**
ID (×4) + GE	43.8	55.2	48.9
ID (×5) + GE	44.7	55.1	**49.4**

Impact of merging several copies of ID training set with GE training and development sets for the Stanford model. F_1 _scores are on ID development data (2N parser only, Core metric). Best performance is achieved when using three to five copies of ID to allow GE data to be used without overwhelming the ID data.

## Discussion

Generally, stacking has led to substantial improvements across the board. There are, however, some exceptions. One is *Binding *events for the GE task. Here the UMass and several reranked Stanford models still outperform the best stacked system (see Table 2). Likewise, for full papers in the Genia test set, the UMass model still does slightly better, with 53.1% F_1 _compared to 52.7% F_1_. This suggests that a more informed combination of our systems (e.g., meta-classifiers) could lead to better performance. For example, a naïve implementation could be to simply avoid stacking for *Binding *events, and within full papers. In order to better understand where the improvements from stacking were coming from, we performed several forms of error analysis. The first examines to what extent projectivity is a factor. The second traces where events proposed by the stacked models originate and explores the novel events generated as a result of stacking.

### Non-projectivity analysis

**Figure 3:**
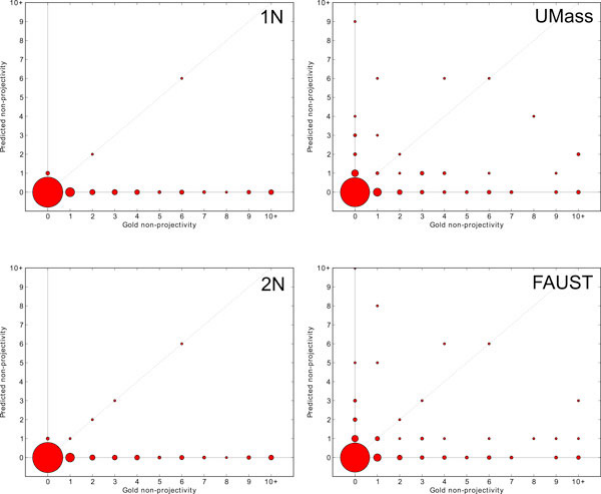
**Predicted non-projectivity**. Accuracy of how well each model predicts the correct number of non-projective structures for each document. Each (*x*, *y*) datapoint compares the number of gold non-projective arcs (*x*) in a document with the number of non-projective arcs in the predicted output for that document (*y*). The area of each point is proportional to the number of documents it covers. A perfect model would put all points along the diagonal *y *= *x *(dashed grey line). The Stanford 1N and 2N decoders, despite having the potential to produce non-projective structures, produce almost completely projective structures. The UMass and FAUST models produce more non-projective structures, though neither is especially precise at predicting the correct amount of non-projectivity. These experiments were performed over the development section of Genia.

One hypothesis is that (non-)projectivity plays a large role in where improvements from stacking occur [27]. This is because the UMass model does not include projectivity constraints while the projective decoders from the Stanford model (1P and 2P) do. Since UMass←Stanford (1P) and UMass←Stanford (2P) perform almost as well as the stacked model with all four decoders (as seen in Table 2), projectivity does appear to be a factor at first. In Figure 3, we show how well models create event structures with the correct amount of non-projectivity. Despite allowing for non-projective structures, the Stanford non-projective (1N and 2N) generally produce completely projective event structures. The projective Stanford decoders (1P and 2P, not shown in Figure 3) naturally predict nearly projective outputs (there are a small number of non-projective structures which are created by the post-processing steps). The UMass and FAUST systems predict non-projective structures much more frequently, though rarely with the correct amount of non-projectivity. Between the UMass and FAUST systems, there doesn't appear to be a large difference in the predicted projectivity. Thus, there is little evidence that the output from the Stanford models greatly influences the projectivity of the FAUST model via stacking.

**Figure 4:**
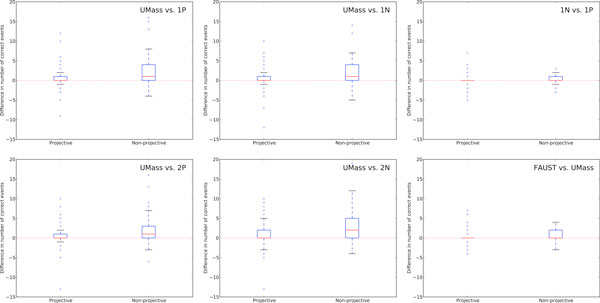
**Differences on projective and non-projective documents**. Document-wise differences in performance (number of predicted events matched with gold) between two models. Positive differences indicate that the first model outperformed the second model. Each chart includes a boxplot for the distributions of these differences for both projective documents (i.e., documents containing no projective arcs) and non-projective documents. Each boxplot shows the median value (solid red line), 25th and 75th percentiles (blue lines), and 1.5 times the interquartile range, (75th percentile to 25th percentile, shown with solid black lines) and outliers (blue plus symbols). In general, larger improvements happen in non-projective documents. The FAUST model performs comparably to UMass on projective documents (the 25th, 50th, and 75th percentiles here are 0). However, on non-projective documents, the differences are generally positive, indicating that this is one class of document that is improved by stacking. These experiments were performed over the development section of Genia. There were 205 projective documents and 54 documents with at least one non-projective arc.

However, in Figure 4, we show that while the predicted amounts of projectivity do not change much between the UMass and FAUST systems, we generally see larger improvements in documents with non-projective structures. These boxplots provide the distributions of differences in accuracy (number of predicted events matched with gold) between two models. Each chart includes a boxplot for the distributions of these differences both for projective documents (i.e., documents containing no projective arcs) and non-projective documents. Each boxplot shows the median value (solid red line), 25th and 75th percentiles (blue lines), and 1.5 times the interquartile range (75th percentile to 25th percentile, shown with solid black lines). For example, the upper left chart shows that the differences between UMass and the Stanford (1P) models on non-projective documents tend to be positive--that is, the UMass system generally performs better on these documents with a median difference of 1 event. In these boxplots, the four Stanford decoders perform similarly to each other with respect to UMass. Despite this, when comparing the 1N and 1P decoders (upper right chart), we see that there is a small improvement on non-projective documents. Performance on projective documents is nearly identical in this case modulo outliers. A similar trend can be seen in the comparison between the FAUST and UMass systems. Putting these two figures together, a larger story can be seen. None of the Stanford models predict high numbers of non-projective structures while the UMass system occasionally overpredicts non-projectivity. When stacked with Stanford decoders, it receives a soft constraint to produce more projective structures.

Despite this, the FAUST system behaves similarly to the UMass system in terms of its predicted projectivity. Thus, while most of the improvements from stacking occur on non-projective sentences, their non-projectivity is mostly unaffected and the improved performance must come from other factors in the structure. That is, the event structures predicted are more accurate but their overall projectivity is not significantly changed.

### Event origin analysis

Next, we investigate the origins of events proposed by the stacked models. Specifically, we aim to answer which base models originally proposed each event (if any) and how many events were novel in the stacked model's output. To perform this analysis, we collect the outputs from the Stanford (2P), UMass, and FAUST models. In the following, we will treat each model as the set of its predicted events. We place each event *e *∈ FAUST in one of four classes:

$$\begin{array}{ll} Only\;2P & e\in \text{FAUST}\;\cap \;(2\text{P}\;-\;\text{UMass})\\ Only\;UMass & e\in \text{FAUST}\;\cap \;(\text{UMass}\;-\;2\text{P})\\ Both & e\in \text{FAUST}\;\cap \;(\text{UMass}\;\cap \;2\text{P})\\ Novel & e\in \text{FAUST}\;-\;(\text{UMass}\;\cap \;2\text{P}) \end{array}$$

**Figure 5:**
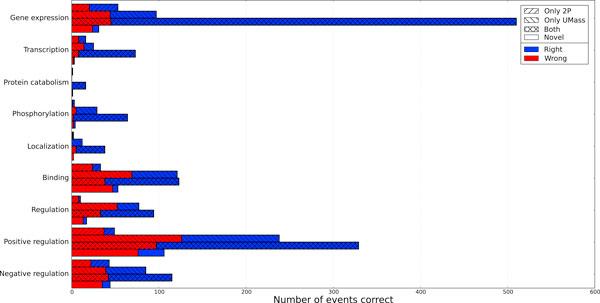
**Origins of events proposed by the stacked model**. For each event in the stacked model's output, we show which model(s) originally proposed the event or whether the event was novel (generated by the stacked model). We group events by their event type and whether they were correct with respect to the gold standard. Event origin (only from the Stanford 2P predictions, only from UMass, from both Stanford 2P and UMass, or novel to the stacking output) is marked by hatching while event correctness is indicated by color. Several observations can be made: Novel events tend to be more incorrect than correct, events originating from both base models have high precision, and *Gene Expression*s events have high agreement between the two base models.

We allow an event to be contained by one of these classes (i.e., *2*) if it *matches *one of the events in the set according to the BioNLP non-approximate recursive scoring rules [2]. For each event type, we show the number of events in each class and the frequency of events that were correct (Figure 5). We can now see that the majority of events are not novel and most originate from the intersection of the Stanford (2P) and UMass models. Furthermore, the novel events are frequently incorrect. There are several compatible explanations. One is that the majority of novel events are in the *Binding *and *Regulation *categories which tend to be more complex and thus harder to predict. Additionally, novel events by definition are ones that haven't been proposed by either model and thus haven't been "vetted" as closely. Contrast this with the high precision of events that come from both base models. Since novel events proved to be unreliable, we experimented with removing them from the output of the stacked model and reevaluated. The net effect is a 0.3% increase in BioNLP F1 to 56.2% for the Genia development section (Table 2). On the Genia test section, this results in a 0.6% improvement over the state-of-the-art result from the FAUST system (Table 1). As expected, the gains come from a 5% boost in precision and a 2% drop in recall. In Figure 5, one can also see that the largest single band consists of *Gene Expression*s proposed by both the UMass and Stanford (2P) models. In general, *Gene Expression*s have very high precision and are easy to recognize. This leads to a large intersection between the models' proposed events.

## Conclusions

We have exploring different methods of model combination for biomolecular event extraction. The leading technique in our experiments, stacking, was used by the FAUST entry to the BioNLP 2011 shared task. By using the predictions of the Stanford models as features of the UMass model, we substantially improved upon both systems in isolation. This helped us to rank first in three of the four tasks we submitted results to. Remarkably, in some cases we observed improvements despite a 5% F_1 _margin between the models being combined.

Stacking and reranking outperform the union and intersection baselines as model combination techniques. By allowing the better performing base model (UMass) to flexibly determine how to incorporate new information from the other base models, the model combination can be done in a more informed and finer-grained fashion.

Our analysis has shed light on where the improvements from stacking originate. While stacking does not improve the model's ability to predict the correct levels of projectivity, it does primarily improve the performance on non-projective documents. Additionally, while stacking can generate novel events, these turn out to be low in precision and ultimately harmful to overall performance.

There are many possible avenues to pursue in the future. While this paper explored stacking the four related Stanford models, using a broader set of base models would certainly improve performance with minimal effort (similar to the experiments run in [1] combining the outputs from the six best systems). Additionally, further attention could be placed on the specific features for stacking. In this study, we explored two feature templates for stacking (non-conjoined and conjoined where non-conjoined ended up performing better) but there is likely a middle ground to allow the stacking model to incorporate the predictions from the stacked model(s) more finely. Finally, it is worth investigating incorporating novel components into the UMass dual decomposition framework, e.g., the maximum-spanning tree component from the Stanford model.

## Competing interests

The authors declare that they have no competing interests.

## Authors' contributions

DM and SR conceived and designed the project with help of MS, CM, and AM. SR implemented the original UMass model. DM and MS implemented the original Stanford model. SR and DM performed the model combination experiments and initial analysis. DM performed the non-projectivity and event origin analyses with help from SR and CM. DM and SR drafted the manuscript with help from CM. CM and AM helped direct the experiments. All authors read and approved the final manuscript.

## Tables

Throughout these tables, the notation *L*←*R *denotes that predictions from model *R *were used as stacking input to model *L*. Note that a previous paper [27] contained incorrect F1 scores from the Stanford system. These have been corrected here.
